# A systematic review protocol on the effectiveness of therapeutic exercises utilised by physiotherapists to improve function in patients with burns

**DOI:** 10.1186/s13643-017-0592-6

**Published:** 2017-10-23

**Authors:** Tapfuma Mudawarima, Matthew Chiwaridzo, Jennifer Jelsma, Karen Grimmer, Faith Chengetayi Muchemwa

**Affiliations:** 1 Rehabilitation Department, Harare Central Hospital, P.O Box ST 14, Southerton, Harare, Zimbabwe; 2 University of Zimbabwe, College of Health Sciences, P.O Box A178, Avondale, Harare, Zimbabwe; 3 School of Health and Rehabilitation Sciences, Faculty of Health Sciences, Observatory, University of Cape Town, Cape Town, South Africa; 4 Stellenbosch University, Cape Town, South Africa

**Keywords:** Strength exercises, Aerobic exercises, Function, Burns, Muscle strength, Physiotherapy

## Abstract

**Background:**

Therapeutic exercises play a crucial role in the management of burn injuries. The broad objective of this review is to systematically evaluate the effectiveness, safety and applicability to low-income countries of therapeutic exercises utilised by physiotherapists to improve function in patients with burns. Population = adults and children/adolescents with burns of any aspect of their bodies. Interventions = any aerobic and/or strength exercises delivered as part of a rehabilitation programme by anyone (e.g. physiotherapists, occupational therapists, nurses, doctors, community workers and patients themselves). Comparators = any comparator. Outcomes = any measure of outcome (e.g. quality of life, pain, muscle strength, range of movement, fear or quality of movement). Settings = any setting in any country.

**Methods/design:**

A systematic review will be conducted by two blinded independent reviewers who will search articles on PubMed, CiNAHL, Cochrane library, Medline, Pedro, OTseeker, EMBASE, PsychINFO and EBSCOhost using predefined criteria. Studies of human participants of any age suffering from burns will be eligible, and there will be no restrictions on total body surface area. Only randomised controlled trials will be considered for this review, and the methodological quality of studies meeting the selection criteria will be evaluated using the Cochrane Collaboration tool for assessing risk of bias. The PRISMA reporting standards will be used to write the review. A narrative analysis of the findings will be done, but if pooling is possible, meta-analysis will be considered.

**Discussion:**

Burns may have a long-lasting impact on both psychological and physical functioning and thus it is important to identify and evaluate the effects of current and past aerobic and strength exercises on patients with burns. By identifying the characteristics of effective exercise programmes, guidelines can be suggested for developing intervention programmes aimed at improving the function of patients with burns. The safety and precautions of exercise regimes and the optimal frequency, duration, time and intensity will also be examined to inform further intervention.

**Systematic review registration:**

PROSPERO CDR42016048370.

**Electronic supplementary material:**

The online version of this article (10.1186/s13643-017-0592-6) contains supplementary material, which is available to authorized users.

## Background

Burn injury is a frequent cause of hospital admission in low-income countries [1–3] and often leads to secondary complications such as disfigurement, contractures and scar tissue formation [1, 2, 4]. Physiotherapy has an important role to play in preventing these impairments and in maintaining and improving functioning and participation in the acute, chronic and rehabilitation phases [5–7]. In resource-constrained contexts, in which access to appropriate therapy may be limited, it is particularly important to identify which rehabilitation and physiotherapy interventions are the most effective in restoration of function.

Therapeutic exercises can be described as bodily movement prescribed to correct an impairment, improve musculoskeletal function or maintain a state of well-being [8]. They have been defined as a range of physical activities that focuses on restoring and maintaining strength, endurance, flexibility, stability and balance [9]. The main goal of therapeutic exercises is to return the injured patient to a fully functioning pain-free state [8, 9]. Therapeutic exercises may include strengthening, endurance, flexibility, balance and coordination exercises [10, 11].

Exercise is beneficial, not only for patients with burns but also for healthy patients [10]. The beneficial effects of exercise are improved cardiovascular health, maintaining a healthy weight, improving bone weight, improvement in self-confidence and social skills [10, 11]. Exercise prescription might be beneficial for patients with burns as they are at an increased risk of bed immobility due to heavy sedation from pain medication, constant wound dressing and bandaging. These factors result in an increase for the demand to exercise due to altered biomechanics, body posture and gait.

The focus of this review is the effectiveness of therapeutic exercises in reducing impairments and functional limitations related to burn injuries. In patients with burns, the damaged tissues may give rise to severe pain [12]. Moreover, pain is one of the most common problems [7, 13] related to therapeutic procedures of restoring function [13, 14] during rehabilitation. Particularly, in children, not only are burns painful, but they also cause distress and anxiety to both the child and the parent [15]. Pain leads to non-compliance, and patients are at high risk of complications of immobility and bed rest. Anecdotal evidence shows that due to lack of resources in low-income countries less effective treatment is done and patients spend more days in the hospital [2]. Potential complications for increased immobility and high admission days include musculoskeletal (decreased muscle strength, decreased endurance, contractures and osteoporosis) and cardiovascular (decreased heart rate, decreased cardiac reserve, orthostatic hypotension and venous thromboembolism) [16].

A search of the Cochrane Review database using keywords “Burns” and “Rehabilitation” or “Physiotherapy/Physical Therapy” or exercise returned one review on the effects of stretch on contractures in people with, or at risk of, contractures [17]. It concluded that stretch on its own was not effective in preventing contractures. There have been studies done on the effect of exercise, but the two [10, 11] systematic reviews could be found in the Cochrane database, the Prospero database or PEDro respectively which examined the effect of exercise did not target exercise prescription for burn patients. There is evidence that exercise might reduce the impact of secondary complications of burns [18], such as muscle weakness and decreased anaerobic capacity, and be as effective as splinting in retaining range of motion of the shoulder after axillary burns [19] and there is thus a need for guidelines, especially those that can be implemented in low-income settings.

Most of the interventions were developed for first world countries [2, 10, 11] but may not be applicable to Africa where most of the countries have resource constraints with regard to health care provision. Examples of this include lack of specialised staff, lack of state of the art equipment and lack of dedicated burn units in medical facilities. Nevertheless, there is also need to implement evidence-based practice in the management of patients with burns in these settings, where ironically, burn injuries are more common [1].

Exercise prescription should be age specific and individualised to meet the different needs of individuals with different level of fitness. It has been shown that exercises might be beneficial for patients suffering from burns [18], but there is lack of guidelines on exercise prescription for these patients.

Burns may have a long-lasting impact on both psychological and physical functioning and thus it is important to identify and evaluate the effects of current and past aerobic and strength exercises on patients with burns. By identifying the characteristics of effective exercise programmes, guidelines can be suggested for developing intervention programmes aimed at improving the physical functioning and, possibly, the health-related quality of life (HRQoL) of patients with burns. The safety and precautions of exercise regimes and the optimal frequency, duration, time and intensity will also inform further intervention.

### Objectives

The broad objective of this review is to systematically evaluate the effectiveness, safety and applicability to low-income countries of therapeutic exercises utilised by physiotherapists to improve function in patients with burns.

- Key review question
  1. What is the efficacy of aerobic and/or strength exercises for individuals with burns, and overall improvement of any measure of outcome?
    P = adults and children/adolescents with burns of aspect of their bodies
    I = any aerobic and/or strength exercises delivered as part of a rehabilitation programme by anyone (e.g. physiotherapists, occupational therapists, nurses, doctors, community workers and patients themselves)
    C = any comparator
    O = any measure of outcome related to physical and psychological functioning (e.g. health-related quality of life, pain, muscle strength, range of movement, fear or quality of movement)
    S = any setting in any country
- Secondary review questions
  2. What precautions and contraindications need to be taken into account during aerobic and strength exercises to individuals with burns?
  3. Are the interventions applicable to physiotherapy delivered in any location in a low-income setting? This will take into consideration issues such as equipment required, staff training, cost, culture and community supports.

The literature identified from the search findings for the key review question will be further reviewed to answer the secondary review questions. Papers will be investigated for additional intervention material:

- For secondary review question 1: articles which describe any attempt to monitor progress for adverse events such as fatigue (measured in any way); excessive pain (measured in any way); or describes a stopping rule (where treatment does not proceed because of safety to the patient, or high likelihood of adverse events occurring from the intervention)
- For secondary review question 2: any article that describes patient samples and interventions relevant to developing countries

## Methods

### Study registration

This systematic review will be written in accordance with the Preferred Reporting Items for Systematic reviews and Meta-Analysis-Protocol (PRISMA-P) guidelines attached as Additional file 1 and has been registered on PROSPERO database (Ref: CDR42016048370).

### Eligibility

In selecting studies, we will apply the following criteria:

- Participants
  Studies of human participants of any age suffering from burns will be eligible, and there will be no restriction on total body surface area (TBSA) affected or duration of intervention to cater for both long- and short-term outcomes in terms of exercise prescription [10]. Both males and females will be considered for the review even though exercise tolerance may differ [11]. Animal studies will be excluded due to their different anatomical structure hence difficulty in exercise prescription. Examples of how studies will be summarised are provided in Table 1.
  Studies with patients suffering from other co-morbidities will be excluded from the study as they might not be able to complete the intervention or their safety might be at risk during standard care procedures [10, 11]. Studies with patients with (1) other neurological conditions and (2) patients unable to comprehend instructions either who are on mechanical ventilators or with decreased level of consciousness will also be excluded. Studies with children who are unable to comprehend instructions and those that are too young or suffering from cognitive disorders will be excluded from this study. Studies on the management of respiratory complications due to inhalational injuries and acute respiratory distress syndrome (ARDS) constitute specialised care and will be beyond the scope of this review. Similarly, studies on the management of psychological effects will not be included in this review, although the impact on HRQoL will be examined. Although burns may also affect the patient and caregivers psychologically, leading to emotional trauma especially where infants or children are involved [2, 20, 21], the management of these conditions is beyond the scope of this review. Studies on the use of virtual reality [22] and behavioural therapy to manage pain have been studied but will not be included in this review as we are focusing on improving function through therapeutic exercises only.

**Table 1:** Characteristics of patients with burns of included studies

Article	Subjects (*n*)	Male (%)	Females (%)	Age (years)	TBSA (%)	Setting
Matthew et al. (2009)	10	0	10	3.2	20	Hospital (inpatient)
Rumbi et al. (2007)	20	15	5	Females 4 males 5.4	Females 10 males 11	Hospital (outpatient)
Mhandi et al. (1995)	25	6	19	Exercise 2 2 control 33	Exercise 40 control 35	Home (home programme)

- Included study designs
  Only randomised control trials (RCTs) that investigate the effects of exposure to any form of therapeutic exercise will be considered for the review.

### Interventions and comparators

Exercises will include any form of exposure to strengthening (resistance) or aerobic (endurance) exercises. RCTs will only be considered if validated outcome measures were measured at least two time points including baseline, follow-up and on completion of the exercise programme. Length of follow-up was not prescribed due to the variability in TBSA. The intervention may be supervised/unsupervised, patient specific in combination or not in combination with standard of care commencing at any period of time either in a hospital setting or outpatient setting. Participants will be compared with another control group or they can act as their own control. Taking cognisance that all ages and TBSA are being considered, it is necessary to limit the study only to RCTs.

### Outcomes

Strength exercise outcome measures might include isometric, isokinetic or isotonic which can either be evaluated through dynamometry, manual muscle testing (MMT) or 3 repetition maximum (3RM). Lean body mass (LBM) could be measured radiographically. Aerobic exercises can be measured by change in heart rate using heart rate monitors and change in lung capacity using VO2 maximal out. Secondary outcomes can be measured through functional activities could include walking and stair climbing which can be used in a low income and are easily applicable in a clinical setting or outpatient department.

Table 2 exemplifies the Participants Interventions Comparison Outcomes (PICO) table.

**Table 2:** Example of PICO table—population, intervention, comparison and outcome

Article	Sample size	Intervention	Comparison	Load, number of repetitions, sets and progression	Frequency and duration	Outcome measure(s)
Strengthening exercises						
Matthew et al. (2009)		Ankle dorsi-flexion exercises with Thera band	Ankle dorsi-flexion exercises	50–80%; 1RM; 2–3 sets; 1 min rest between sets	3× per week for 2 weeks	MMT (muscle strength)
Aerobic exercises						
Rumbi et al. (2007)		Cycling	Nil cycling	70% of VO2 max; 5 × 5 mins bouts; 2 min rest between each bout	3× per week × 5 weeks	VO2 max; HR and RR
Combination programmes						
Mhandi et al. (1995)		Muscle strengthening, dynamic balance	Nil dynamic balance	Knee muscle strengthening, dynamic balance through ball transfer from one hand to another × 5 mins	6× per week for 2 weeks	Gait and posture

### Language

We will only consider full text articles published in English.

### Information sources

#### Search strategy

A comprehensive search will be conducted at the University of Cape Town library during the period of July 2017 to August 2017. All accessible bibliographic databases of published research reports will be assessed. All databases will be searched from 1990 to date. The electronic databases will include PubMed, CiNAHL, Cochrane library, Medline, Pedro and OT seeker, EMBASE, PSYCH INFO and EBSCOhost. Manual searches of reference lists of included articles will be employed.

The terms in the title (“physiotherapy” OR “physical therapy” OR “rehabilitation” OR Occupational therapy) AND (“burns” OR “burns patient” OR “patient with burns”) AND (“exercises” OR “therapeutic exercises”). Outline in Table 3 is an example of how literature will be searched in CINAHL.

**Table 3:** Search strategy

Keyword	Alternative words
Physiotherapy	physical therapy OR rehabilitation OR occupational therapy OR physical medicine OR physiatrist
Burns	patients with burns or burns patient
Muscle strength	muscle bulk OR muscle size OR lean muscle strength
VO2 max	respiratory rate OR heart rate
Strengthening exercises	Resistance exercises
Aerobic exercises	Endurance exercises

### Study records

#### Data management

Search results will be merged using reference management software (Covidence) which is data management software. The electronic searches will also be saved to the researcher’s PUBMED account. The principal researcher will create a shared DROPBOX folder to facilitate collaboration among reviewers and to save the online versions of the articles and electronic search strategy. Summaries of all the searches are to be printed and are to be used as physical backup for the screened articles.

#### Selection and data collection of studies

Two reviewers Matthew Chiwaridzo (MC) and Tapfuma Mudawarima (TM), both physiotherapists, will independently search the databases and screen the titles and abstracts for eligibility. Title and abstracts will be examined to remove irrelevant reports and full text of potentially relevant reports will be obtained; multiple reports of the same study will be linked to minimise bias of duplicate publication. The two reviewers will assess full text reports for compliance with the eligibility criteria, and correspondence will be done with authors to clarify study eligibility, where necessary.

In case of disagreement, arbitration by a third reviewer Jennifer Jelsma (JJ) will be carried out. Full text which meets the eligibility criterion will be assessed for risk of bias and data analysis/synthesis independently by TM. The reviewers will also manually search the references for articles to include in the data extraction.

### Outcomes and prioritisation

#### Outcomes

For this review, outcome measures for strength exercises will include manual muscle testing (MMT) and lean body mass (LBM) or increase in muscle bulk and for aerobic exercises will include VO2max and HR. Secondary outcomes will include sit to stand, gait, stair climbing and HRQoL measured by the burn-specific health scale or generic HRQoL scales. Patient reported outcomes will be prioritised in the discussion.

#### Assessment of risk bias (or “quality”) individual studies

The Cochrane Collaboration tool for assessing the risk of bias [23] in experimental studies will be used to assess the methodological quality of the included studies. The PRISMA reporting standards will be used to guide the review report (http://www.prisma-statement.org/Extensions/Protocols.aspx).

#### Best evidence synthesis

A narrative synthesis of the findings from the included studies will be provided due to the likely heterogeneity of the intervention and outcome measures. The Template for Intervention Description and Replication (TIDieR) checklist will be used to describe components of the exercise interventions which are reported in each included study [24]. The exercise interventions will be described in subgroups of outcome measures of strength, aerobic and functional measures, and data within individual studies such as patient’s population and interventions will be described in a narrative summary. Information on adherence to exercises, compliance monitoring, resources used and costs incurred in the delivery and receipt of services will be extracted, if available. If pooling is possible from the intervention data (as a whole, or in subsets of the included studies), meta-analyses will be considered. Revman software will be used for this [25].

## Discussion

As far as we are aware, this will be the first review of experimental evidence related to aerobic exercises and strength training delivered in any setting, for patients of any age, suffering from burns to any part of the body. This review will provide an answer to the question of efficacy for this type of intervention for burn patients. Subsequent analysis of intervention information reported in the included literature will provide previously unavailable information on the elements of the interventions, whether specific elements are related to evidence of significant benefit, and whether best practice can be determined for the delivery of aerobic exercises and strength training for patients with burns. Specific information on safety measures that have been put in place to prevent adverse events during aerobic exercises and strength training for patients with burns will be highlighted. Where information is available on this intervention that is relevant to burn patients in low-income countries, the authors will compile evidence-informed guidance for the safe delivery of aerobic exercises and strength training in these settings.

## Additional file

Additional file 1: PRISMA-P 2015 Checklist. (DOCX 34 kb)

## Abbreviations

3RM: 3 repetition maximum
ARDS: Acute respiratory distress syndrome
CSP: Chartered Society of Physiotherapy
MMT: Manual muscle testing
PICO: Participants Interventions Comparison Outcomes
PRISMA-P: Preferred Reporting Items of Systematic review and Meta-Analysis-Protocol
RCTs: Randomised controlled trials
RoM: Range of motion
TBSA: Total body surface area
